# Critical Analysis of Hypothesis Tests in Federal Information Processing Standard (140-2)

**DOI:** 10.3390/e24050613

**Published:** 2022-04-27

**Authors:** Elena Almaraz Luengo, Marcos Brian Leiva Cerna, Luis Javier García Villalba, Julio Hernandez-Castro, Darren Hurley-Smith

**Affiliations:** 1 Group of Analysis, Security and Systems (GASS), Universidad Complutense de Madrid, 28040 Madrid, Spain; ealmaraz@ucm.es (E.A.L.); marcolei@ucm.es (M.B.L.C.); 2 School of Computing, University of Kent, Canterbury CT2 7NZ, UK; jch27@kent.ac.uk; 3 Information Security Group, Royal Holloway University of London, Egham TW20 0EX, UK; darren.hurley-smith@rhul.ac.uk

**Keywords:** correlation, Dieharder, ENT, FIPS 140-2, independence, mutual information, NIST SP 800-22, *p*-value, randomness, statistic, TestU01

## Abstract

This work presents an analysis of the existing dependencies between the tests of the FIPS 140-2 battery. Two main analytical approaches are utilized, the first being a study of correlations through the Pearson’s correlation coefficient that detects linear dependencies, and the second one being a novel application of the mutual information measure that allows detecting possible non-linear relationships. In order to carry out this study, the FIPS 140-2 battery is reimplemented to allow the user to obtain *p*-values and statistics that are essential for more rigorous end-user analysis of random number generators (RNG).

## 1. Introduction

The generation of random or pseudo-random sequences is crucial for scientific, cryptographic, and even entertainment purposes; from the generation of random variables [1,2], for mathematical or analytical purposes (for example, [3,4,5,6], and others) to applications in information and communication technologies [7,8,9], and image encryption [10] in medicine [11] among others.

The degree of randomness required by a given application can vary. Sequences may need to be reproducible (seeded RNGs produce these for simulations) or ‘truly’ random (e.g., cryptographic keys). The shared requirement is that one has some way of verifying that a RNG is sufficiently random for the target application. For the purpose of verifying the goodness of generated sequences, different statistical tests are used, which, to a given degree of confidence (α level), inform users whether the sequences can be used in such systems. Such tests may also be used to profile RNGs so that their flaws may be identified, reported, and rectified. Statistical tests of randomness are implemented in a variety of software languages, with some (FIPS 140-2 in particular) implemented in both FPGA and ASIC to provide rapid in-line testing of the RNG ouput (e.g., so-called total failure tests in hardware RNGs). Some of the best-known batteries in the literature are NIST SP 800-22 [12], TestU01 [13], Dieharder [14], ENT [15], and FIPS 140-2 [16] among others [17].

To successfully use a statistical test (especially a group of them, or battery), one must be aware of the attributes tested, rigor, and duration of the tests. The application of many different hypothesis tests, to ensure a thorough analysis of various traits of a sequence (such as independent and identical distribution if such is desired), may take significant computational time: hours or possibly days, even on high-end systems (e.g., 64-bit 3.6 GHz+ 8-core CPU 32 GB RAM DDR4 3066 MHz+, for the context of high-end at the time of writing). For this reason, one of the current lines of research is the analysis of the interrelationship that may exist between the different tests that make up the batteries in order to, if there is one, discard any of the tests which duplicate results with little additional value. One of the most widely used approaches is the analysis of linear correlations between the different obtained *p*-values ([18,19,20], among others) or even between the statistics directly (see, for example, the reasoning about this issue in [21]). The most popular correlation measure used in this approach is Pearson’s correlation coefficient. Given two random variables *X* and *Y*, this coefficient is defined as

$$\rho_{X,Y}=\frac{\mathrm{cov}(X,Y)}{\sigma_{X}\sigma_{Y}}$$

where $\sigma_{X}$ and $\sigma_{Y}$ are the standard deviations of *X* and *Y*, and cov(X,Y) is the covariance between them. Its value belongs to the interval [−1,1] and if |ρ| has a value close to 1, it indicates a high linear dependence, and the sign informs if the dependence is direct or inverse. If |ρ| has a value close to zero, it indicates the lower (linear) dependence between the variables. For random samples $X=\left\{x_{1},\ldots ,x_{n}\right\}$ and $Y=\left\{y_{1},\ldots ,y_{n}\right\}$, the sample correlation $r_{X,Y}$ is defined as

$$r_{X,Y}=\frac{\sum_{i=1}^{n}\left(x_{i}-\bar{x}\right)\left(y_{i}-\bar{y}\right)}{\sqrt{\sum_{i=1}^{n}{\left(x_{i}-\bar{x}\right)}^{2}}\sqrt{\sum_{i=1}^{n}{\left(y_{i}-\bar{y}\right)}^{2}}}$$

where $\bar{x}$ and $\bar{y}$ are the arithmetic means of *X* and *Y*, respectively. The drawback of this approach is that the analysis focuses only on the study of linear relationships, omitting other possible (non-linear) relationships. Because of this, the analysis of interrelationships through mutual information (*MI*) [22] was recently proposed, as this measure allows for the detection of non-linear relationships between variables. Mutual information is always used to evaluate the “amount of information" obtained about one random variable when given the other random variable. If *X* and *Y* are continuous random variables with values in $S_{x}$ and $S_{y}$, *MI* is defined as

$$MI=\int_{S_{x}}\int_{S_{y}}p\left(x,y\right)\;\log\left(\frac{p(x,y)}{p(x)p(y)}\right)\;dxdy$$

where p(x,y) is the joint distribution of *X* and *Y*. If *X* and *Y* were discrete, then:

$$MI=\sum_{i=1}^{n}\sum_{j=1}^{m}p\left(x_{i},y_{j}\right)\;\log\left(\frac{p(x_{i},y_{j})}{p\left(x_{i}\right)p\left(y_{j}\right)}\right)$$

where p(x,y) is the discrete joint distribution of *X* and *Y*. Some of the areas in which *MI* has been applied are, for example, in lip reading [23], medical image segmentation [24], signal analysis [25], information theory [26] or clustering analysis [27], among others. For more details about mutual information, see [28].

In this research, we analyze the FIPS battery in detail. The design of this test battery, in terms of its output and analytical value, is one of the main issues when considering FIPS 140-2 as a means of determining whether an RNG is appropriate or safe to use in a given context. In this case, in contrast to NIST SP 800-22, for example, the implementation only provides the user with information on whether or not a sequence passes the applied test, but does not give more details. This prevents users from making judgments based on the statistical data generated by those tests, reducing a complex analysis to a Boolean pass/fail parameter that defies analysis without the use of more verbose tests. In this research, the battery is re-implemented to provide the user with a wider range of statistics, and the results are analyzed both from the point of view of Pearson’s correlation and from the point of view of the mutual information measure.

With the new implementation (whose code we present in this paper), the user can apply the battery to real data and obtain the *p*-values associated to the different tests that form the battery. This allows the user to check the sequences obtained by different generators and to decide if the generated sequences can be considered of good quality or if they need to be improved. The presentation of the *p*-values allows the user to have a statistical measure of the result obtained with each of the tests and to perform more in-depth studies related to the threshold that could be considered for the α level of a test that would change, for example, from rejecting a hypothesis in the test to having no evidence to do so with that α.

This paper is organized as follows: in Section 2, the re-implementation of FIPS test battery is explained; in Section 3, the materials and methods used in our analysis and an analysis of the independence of the test in the battery is performed; finally, in Section 4, the conclusions of the study are given.

## 2. FIPS Test Battery and the New Implementation

The FIPS 140-2 [16] battery is the successor of the FIPS 140-1 standard. It provides the same tests as 140-1, but with updated and stronger conditions for passing, with revised confidence intervals for all tests. It is a battery that, despite its limitations, is widely used by various manufacturers as the standard due to its speed and understandable (if cursory) output. An interesting study about this battery can be found in [29]. There is a new standard, FIPS 140-3 (https://csrc.nist.gov/publications/detail/fips/140/3/final, accessed on 15 March 2022), published in 2019. FIPS 140-3 does not implement statistical tests. However, as far as RNG testing is concerned, it focuses on entropy source modeling. Despite the existence of this version, FIPS 140-2 is still used by manufacturers when onboarding new RNGs, or as a start up/procedural check of sequences with expectations of randomness. FIPS 140-2 results are also frequently used in marketing materials for RNG hardware.

The rng-tools module for Linux includes an implementation of FIPS 140-2 (in rngtest). However, this implementation is not suitable for the purposes of this research for two reasons: (i) it works only with a fixed number of bits (20,000 bits), and (ii) it only tells us if a sequence has passed the tests or not and it does not provide a statistic or a *p*-value. This work re-implemented these tests to overcome these limitations. The code was designed in Python and can be found in Appendix A.

With the new implementation, the tests work for any sequence size (though sequences smaller than 20,000-bits will not produce reliable results), and return both internal test statistics and the *p*-values calculated over those stats. The first three are broadly the same, except that instead of comparing the statistics with a range, statistical tests are performed (a binomial test in Monobit, and a Chi-square goodness-of-fit test in Poker and Runs). However, we need to make further changes to the last two tests for two reasons:

- In both cases, a value is not compared to a range, but directly fails the test if certain requirements are met (streaks of more than 25 bits in Long Run, two consecutive equal blocks in Continuous Run). This can be improved by calculating the probability of test-specific conditions occurring (runs of bits, repeat sequences, etc.), counting the number of times these cases occur, and performing a binomial test.
- The probability of fail conditions occurring is too low for the sequence size tested (at most $10^{7}$). This makes the expected frequency of these conditions almost always 0 so the *p*-value is, most of the time, also 0. As the required sequence size is too large to run the battery in a reasonable time, we changed the original tests to increase the probability: the minimum length in the Long Run test goes from 26 bits to 8, and the block size in the Continuous Run test goes from 32 bits to 4. We leave it as future work to optimize the battery (or a possible C implementation) to repeat the tests with larger sequences and thus address the original tests.

### 2.1. Monobit Test

It consists of counting the number of ones, $c_{1}$, in a sequence. On the original battery, the test is passed if this number is between 9725 and 10,275. Adapting this test is quite simple: to measure the randomness of a sequence, a binomial test must be applied. The number of data is *n* (length of the sequence), and the expected value is $c=\frac{n}{2}$. The resulting *p*-value will inform about how close to *c* the statistic $c_{1}$ is.

### 2.2. Poker Test

The sequence is divided into 4-bit blocks. For each block, there are $2^{4}$ possible values; now, it is counted how many times each of them occurs. In the original implementation, the following formula is applied to the frequencies $f_{i}$:

$$X=\frac{16}{5000}\sum_{i=0}^{15}f_{i}^{2}-5000$$

and it is checked if X∈[2.16,46.17]. This formula is actually a Chi-square goodness-of-fit test [30]. In the experimental case, the observed values are the frequencies $f_{i}$, and the expected values are the frequencies $e_{i}=e=5000/16$, so

$$\sum_{i=0}^{15}\frac{{\left(f_{i}-e\right)}^{2}}{e}=\frac{16}{5000}\sum_{i=0}^{15}f_{i}^{2}-5000$$

Now it is possible to scale the test to sequences of size *n*, taking into account that there are n/4, so $e_{i}=e=\left(n/4\right)/16=n/64$. Then, the statistic is

$$X=\sum_{i=0}^{15}\frac{{\left(f_{i}-e\right)}^{2}}{e}=\frac{64}{n}\sum_{i=0}^{15}f_{i}^{2}-\frac{n}{4}$$

which follows a Chi-Square distribution and the *p*-value is: p=P(z>X).

### 2.3. Runs Test

A run is a set of consecutive elements in the sequence (in this case consecutive 0 s or 1 s). This test calculates all the runs in a sequence and classifies them according to their element (0 or 1) and the size in bits: 1, 2, 3, 4, 5, and 6+ (6 or more). In the original battery, the sequence passes this test if the number of elements in each category is in the range shown in Table 1.

The ranges are the same for runs of 0 s and runs of 1 s. It is possible to perform a Chi-square goodness-of-fit test, where the observed values are $s_{i}^{j}$, with i∈S={1,2,3,4,5,6+} and j∈{0,1}. Under the hypothesis of randomness, the expected number of runs of zeros and ones must be the same for each size, that is, $e_{i}^{0}=e_{i}^{1}\;\forall i\in S$, so it will be considered, without loss of generality, only the streaks of 1 s. Let k∈S and n>>k, with *n* being the sequence length.

- If the run does not appear at the beginning or at the end of a sequence, we must set k+2 bits: the *k* ones of the run and the two zeros that delimit the beginning and end of the run.
  $$\cdots 0\overset{k}{\overbrace{1\cdots 1}}0\cdots$$
- If the run appears at the beginning or end of the sequence, then we only need one zero to delimit the run, so we need to set k+1 bits:
  $$\overset{k}{\overbrace{1\cdots 1}}0\cdots$$
  $$\cdots 0\overset{k}{\overbrace{1\cdots 1}}$$

By linearity, the expected number of runs is $e_{k}^{1}=\left(n-k-1\right)p^{k}{\left(1-p\right)}^{2}+2p^{k}\left(1-p\right)$, with *p* being the probability that a bit in the sequence is 1. In this case, p=1/2, so $e_{k}^{1}=\left(n-k+3\right){\left(1/2\right)}^{k+2}$. Similarly, $e_{k}^{0}=e_{k}^{1}$. In addition, the total expected number of runs is [31]: $e_{T}=\left(2n_{0}n_{1}/\left(n_{0}+n_{1}\right)\right)+1$ where $n_{0}$ is the number of zeros and $n_{1}$ is the number of ones. Under the hypothesis of randomness, $n_{0}=n_{1}=n/2$ so $e_{T}=\left(\left(n^{2}/2\right)/n\right)+1=\left(n/2\right)+1$ and $e_{6+}=e_{T}-\sum_{i=1}^{5}\sum_{j=0}^{1}e_{i}^{j}=\frac{n}{64}+\frac{7}{16}$. Then $e_{6+}^{0}=e_{6+}^{1}=e_{6}/2=\left(n/128\right)+\left(7/32\right)$. Now it can be performed the goodness-of-fit test:

$$R=\sum_{i\in S}\sum_{j=0}^{1}\frac{{\left(s_{i}^{j}-e_{i}^{j}\right)}^{2}}{e_{i}^{j}}$$

### 2.4. Long Run Test

Originally, a sequence fails the test if it has a run of length greater than 25 bits. The simplest idea would be to perform a binomial test with the expected value number of runs of size 26 or more. However, this amount would be too small. As the size of the sequences in our experiments is not large, they have an effectively zero probability of 0 runs of this size or more (the minimum size in our experiments is n=1 MB $=8\times 10^{6}$ bits, in that case $e_{26+}≃0.619$). This problem was not significant in the original test (which only determined whether the sequence passed or not), but it affected our experiments, as this translates into almost always obtaining the same *p*-value for high-order runs. There are two solutions to address the problem: (i) working with much larger sequences, or (ii) altering the original test, causing it to fail with runs of less length. This solution was tested with runs of size 8 bits or more and gave a greater range of expected quantities. This is, therefore, not truly reflective of the original test, but is used to provide a representative and meaningful statistic for use in a subsequent binomial test. In this research, solution (ii) was taken. The number of runs of size 8 or more was used as the expected value for the binomial test. In that case, $e_{8+}=e_{T}-\sum_{i=1}^{7}\sum_{j=0}^{1}e_{i}^{j}=\frac{n+2^{7}-6}{2^{8}}$. If n=1 MB, then $e_{8+}≃31,250.48$.

### 2.5. Continuous Run Test

The original test divides the sequence *u* into *N* blocks of 32 bits, and associates to each block a real number in (0,1), using the transformation

$$b_{1}\ldots b_{32}\to \frac{\sum_{i=1}^{32}2^{32-i}\cdot b_{i}}{2^{32}}$$

The test fails if a run is found. This is equivalent to say that two consecutive blocks are equal without the need to transform each block into a real number.

On this basis, the *p*-value is calculated. Again, the possibility of performing a binomial test (with expected value *e* being the number of times two consecutive blocks are equal) is presented. There are N−1 pairs of consecutive blocks, and the probability that two blocks are equal is $p=1/2^{32}$ so $e=\left(N-1\right)\cdot p=\left(N-1\right)/2^{32}$. As in the previous test, this number is almost always 0 if the sequence is not sufficiently large. It is decided to alter the test (in this, 4-bit blocks are used, which offer a greater variety of equal-block pairings within a given sequence to analyze and characterize).

## 3. Analysis of the Independence of the Tests in FIPS Battery

Two machines are used for this analysis:

- Windows machine with specifications:
  - Operating System: Windows 10 (64-bit)
  - CPU: AMD Ryzen 3700XT (3.6 Ghz, 8 cores, 16 threads)
  - RAM: 64 GB
- Linux machine with specifications:
  - Operating System: Debian (64-bit)
  - CPU: Intel Core 6200U (2.3 Ghz, 2 cores, 4 threads)
  - RAM: 8 GB

In general, the Windows machine is used for the main calculations, as it is more powerful, reserving the Linux machine for the generation of sequences. The elements that are selected for the experimentation are shown in Table 2. As the results are not significantly different when changing the sequence size or the generator, it is shown the case of sequences of $10^{7}$ bits, generated with dev/urandom. We work with Pearson’s correlation coefficient and mutual information. For both measures, running a single experiment is not ideal: recall that the resulting *p*-values are uniformly distributed in (0,1) so that for an α significance, the probability of failing the test is α. Therefore, we carry out 100 different tests for each pair of tests (both Pearson’s correlation and mutual information), and we execute a Kolmogorov–Smirnov (K-S) test on each set of 100 *p*-values, thus we have a reliable measure of their uniformity.

**Figure 1:**
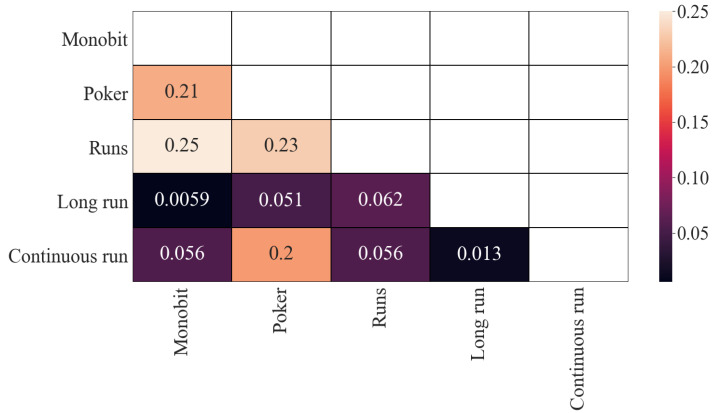
Pearson’s correlation (*p*-values): results.

In Figure 1 are represented the results corresponding to Pearson’s correlation of the obtained *p*-values. As can be seen, there is some correlation between (i) the Poker and Monobit tests, (ii) Monobit with Runs tests, (iii) Poker and Runs tests and (iv) Poker and Continuous Run tests. In all cases, the correlation is similar (around 0.2). The rest of the correlations are, in principle, low, although they are significantly lower between Long Run and Monobit, and between Continuous Run and Long Run.

**Figure 2:**
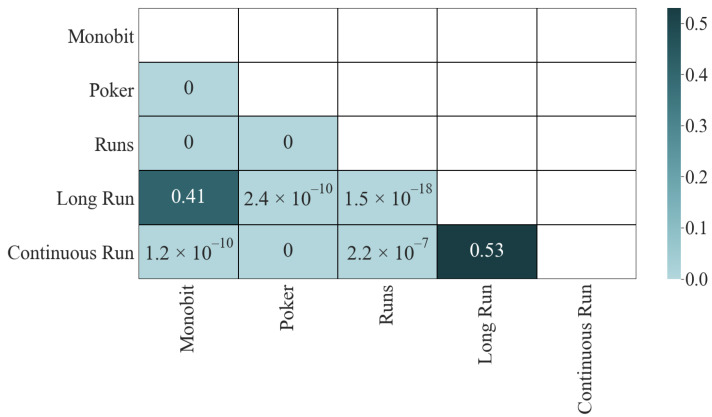
K-S test results.

Let us turn to the significance matrix (Figure 2). The results of the K-S test give us interesting information; only the two pairs with the lowest correlation passed the test: Long Run and Monobit (0.41) and Continuous Run and Long Run (0.53). The other four pairs, which initially did not have a high correlation, did not pass the test: $1.2\times 10^{-10}$ for Continuous Run and Monobit, $2.4\times 10^{-10}$ for Long Run and Poker, $1.5\times 10^{-18}$ for Long Run and Runs, and $2.2\times 10^{-7}$ for Continuous Run and Runs, which is an indicator that they share dependencies. Finally, the four most correlated pairs fail the test with a *p*-value of ϵ in all cases.

**Figure 3:**
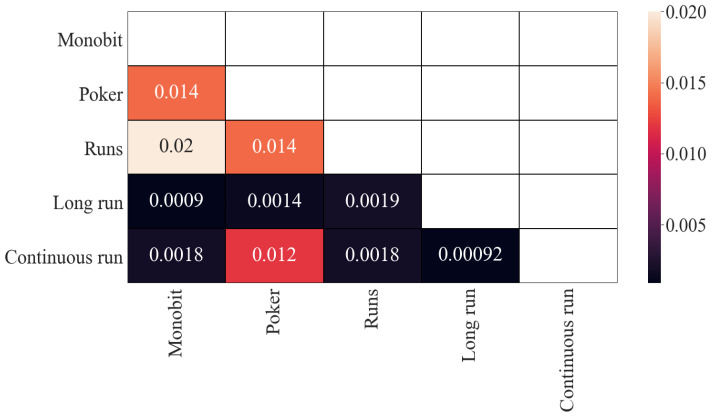
Mutual information (*p*-values): results.

With regard to the results of mutual information, the obtained results are similar (see Figure 3) although on a different scale. The previous four correlated pairs remain correlated, with values around 0.013, more than 10 times less than in Pearson’s correlation results. That could be an indicator that the dependence in these pairs is mainly linear. In the same way, the two least-correlated pairs remain (around 0.001), although this time they are closer to the other values (around 0.0016).

**Figure 4:**
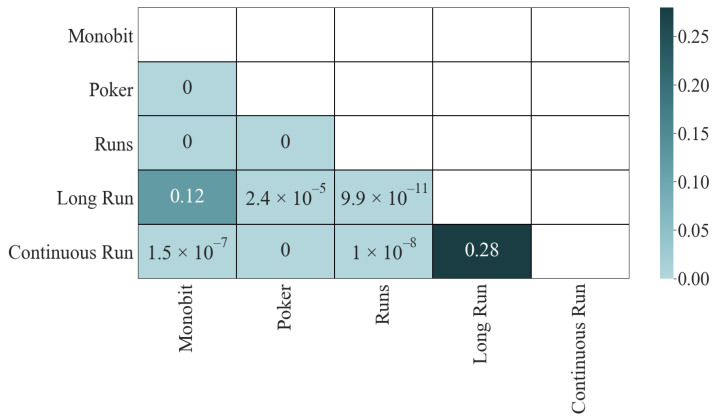
Mutual information (*p*-values): K-S.

The results of the K-S test do not vary concerning those of the correlation (see Figure 4): the four most dependent pairs have a *p*-value of ϵ, the two least correlated pass the test, and the other four fail it, although not in such an extraordinary way (between $2.4\times 10^{-5}$ and $9.9\times 10^{-11}$).

**Figure 5:**
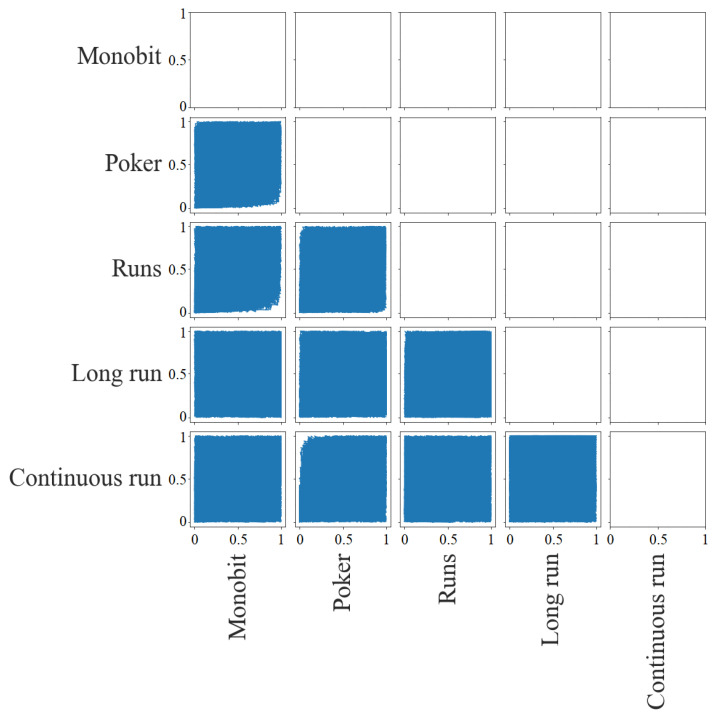
Dispersion matrix (*p*-values).

Figure 5 shows the dispersion matrix of the *p*-values. It is possible to detect the largest correlations between tests. There is a little deviation in the lower right corner for the pairs Poker with Monobit, Runs with Monobit, and Runs with Poker, that is, there are no cases in which the first of the tests has a low *p*-value and the second has a high *p*-value. As for the pair between Continuous Run and Poker, this deviation is in the upper left corner. There are no cases in which the first has a high *p*-value and the second has a low *p*-value. In the rest of the pairs, it is not possible to detect dependencies.

**Figure 6:**
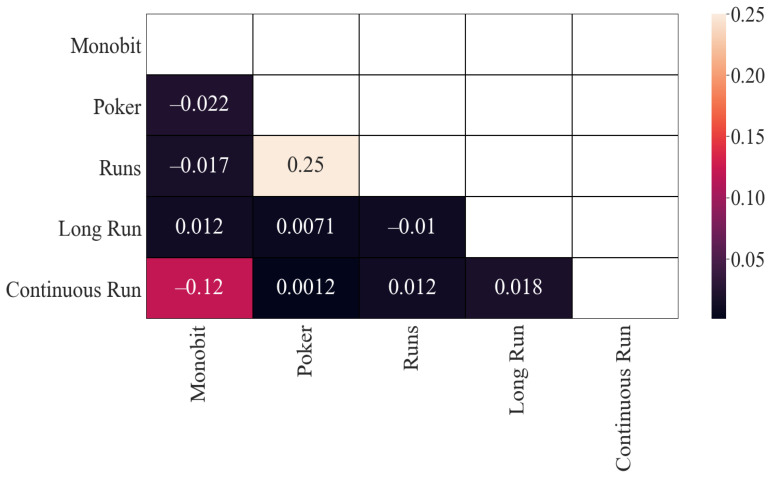
Pearson’s correlation (statistics): results.

In addition, the study was developed on the values obtained for the statistics output by FIPS 140-2 tests for 100 random sequences. This approach was adopted to provide a more rigorous analysis than that provided by *p*-value comparisons alone. While *p*-values inform us of a test’s result, given various input, and can allow one to judge whether two tests consistently report the same outputs, it is only a deeper analysis of the statistical basis for these *p*-values that allows us to determine if the correlation is due to test characteristics, or the influence of tested sequences. In Figure 6, the results with the Pearson’s correlation applied to the statistics are represented. This matrix is very different from that of the *p*-values. Of the four most correlated pairs, only one remains (Runs with Poker) with a similar value (0.247889). Only one more pair has a prominent correlation: Continuous Run with Monobit (−0.124682). The rest of the correlations, in principle, do not seem to be very high (around 0.01).

**Figure 7:**
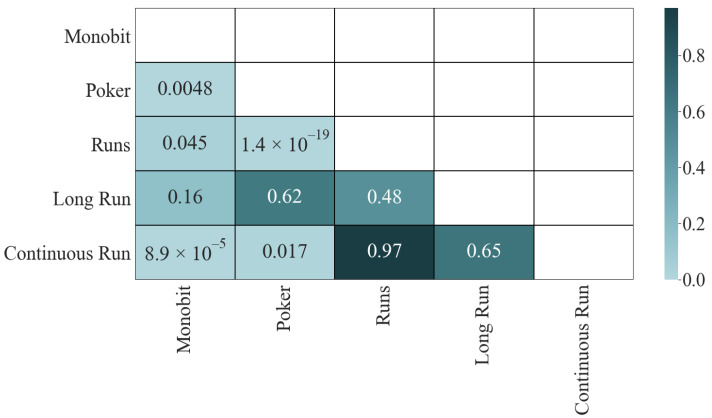
Pearson’s correlation (statistics): K-S.

These results remain in the K-S test (Figure 7), where only these two pairings fail the test. The rest pass (although some were borderline, such as Poker with Monobit (0.0048)).

**Figure 8:**
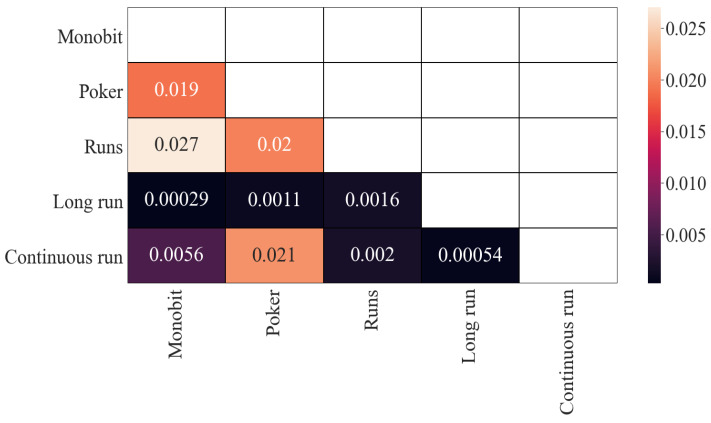
Mutual information (statistics): results.

Results related to the mutual information measure can be seen in Figure 8. Unlike what we saw in Pearson’s correlation, these results are more consistent with those obtained for the *p*-values. In general, the values obtained remain in the same line, distinguishing the three usual cases. Perhaps the most prominent variation is in the pair between Continuous Run and Monobit (from 0.001801 to 0.005572, three times more, but still low).

**Figure 9:**
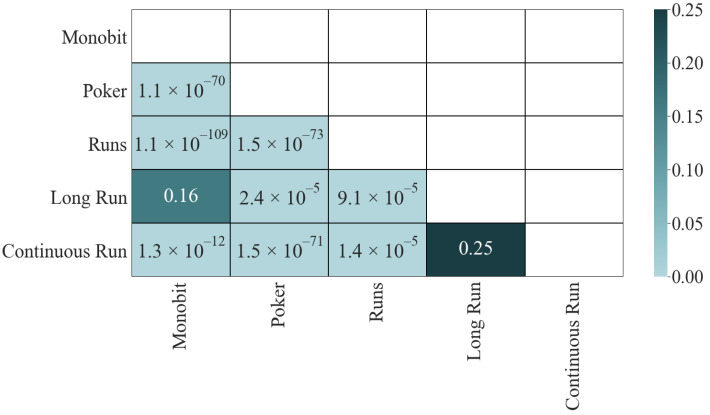
Mutual information (statistics): K-S.

The K-S matrix (Figure 9) corroborates these results. As with *p*-values, only two pairs pass the test. The main change concerning those results is that the pairs that do not pass the test have a higher *p*-value than before (for example, the four most correlated pairs go from ϵ to values around $10^{-70}$), but they are still invalid. These results show us that mutual information is more resistant to changes and that it is capable of giving a general measure of independence between tests.

**Figure 10:**
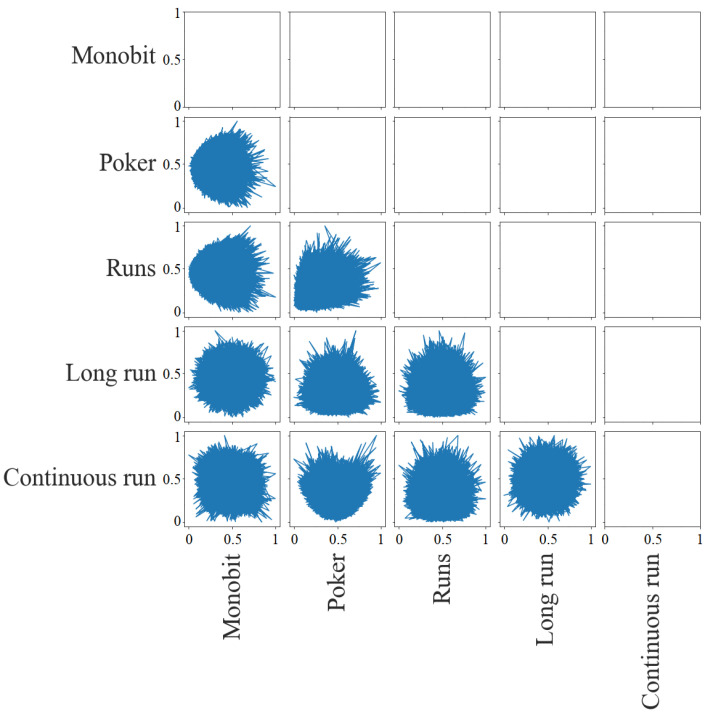
Dispersion matrix (statistics).

Figure 10 provides the dispersion matrix for the statistics study. The most appreciable graphical dependencies are those between (i) Runs with Poker (they have a distribution with a deviation toward the lower right corner that is, they share low *p*-values); (ii) Poker and Runs with Monobit (their distribution has a deviation toward the left, so Monobit obtains low *p*-values when the others have *p*-values around the mean) and (iii) Continuous Run with Poker (its distribution has downward deviation, so Continuous Run obtains low *p*-values when Poker has p-values around the mean). There is also a slight downward deviation, like the last case, between Long Run and Poker, Long Run and Runs, and Continuous Run and Runs.

## 4. Conclusions

In this work, we carried out a study of the linear and non-linear dependencies between FIPS 140-2 battery tests. In order to carry out this analysis, it was necessary to re-implement the battery in such a way as to provide the user with the *p*-values and statistics resulting from the application of different hypothesis tests within the battery. The original tests (as implemented in the rngtools rngtest suite) only provide a Boolean pass or fail output, and so this re-implementation is vital for the success of this analytical work.

As for the analysis of *p*-values, we were able to verify that the results derived from the analysis using Pearson’s correlations and mutual information are similar, although measured on different scales, with those relating to mutual information being more than 10 times lower than those of Pearson’s correlation. This suggests that the existing relationships in this battery are fundamentally linear. With regard to the analysis of the statistics, we were able to verify that the mutual information measure is more resistant to changes and that it is capable of providing a general measure of independence between tests.

The most important interrelationships are between the Poker, Runs, and Monobit tests, with dependencies on each other. If required to select a single test (for the purposes of streamlining the test battery while retaining meaningful output), it would be Monobit, as Poker also has dependencies on Continuous Run, and the Runs test is somewhat more complex. In addition, of the three, it is the one who has the lowest correlation with Long Run.

We are left with three tests (Monobit, Long Run, and Continuous Run), but there is still a dependency (not as big as the first ones, but significant) between Continuous Run and Monobit. If the ultimate objective is the elimination of redundancies in a battery, some of these two tests should be eliminated, but we consider that more studies with the Continuous Run test should be done. Recall that this test is not the original one and depends on a parameter (block size). It is left as future work to test other versions of this test to see if any of them are independent of Monobit.

## Figures and Tables

**Table 1:** Ranges and lengths in runs test.

Length	Range	Length	Range
1	2343–2657	4	251–373
2	1135–1365	5	111–201
3	542–708	6+	111–201

**Table 2:** Information and parameters of the experiments.

Number of Sequences	Size (Bits)	Generator	Significance
		dev/urandom	
$10^{4}$	$10^{5}$	CryptGenRandom()	0.001
	$10^{6}$	python.secrets()	
	$10^{7}$	qRNG	
